# Challenges of Primary Care Medicine in a Tertiary Care Setting—The Case of Primary CMV Infection Compared to Primary EBV Infection: A Retrospective Cohort Study

**DOI:** 10.3389/fmed.2022.880610

**Published:** 2022-06-14

**Authors:** Samuel Etienne, Karoline Leuzinger, Hans H. Hirsch, Michael Osthoff

**Affiliations:** ^1^Division of Internal Medicine, University Hospital Basel, Basel, Switzerland; ^2^Clinical Virology, Laboratory Medicine, University Hospital Basel, Basel, Switzerland; ^3^Department of Biomedicine, University of Basel, Basel, Switzerland; ^4^Department of Clinical Research, University of Basel, Basel, Switzerland

**Keywords:** cytomegalovirus, Epstein-Barr virus, infectious mononucleosis, smarter medicine, primary care medicine, tertiary care center

## Abstract

**Background:**

In the immunocompetent adult primary cytomegalovirus (CMV) infection may present as prolonged febrile illness or may resemble infectious mononucleosis. Hence, establishing a diagnosis of primary CMV infection may be challenging, in particular in the hospital setting.

**Methods:**

We performed a retrospective analysis of all immunocompetent patients treated at a tertiary care center in Switzerland over a 5-year period in whom a diagnosis of primary CMV infection was established. We assessed their demographic, clinical, and laboratory characteristics and compared them to patients with a diagnosis of primary Epstein-Barr virus (EBV) infection during the same period.

**Results:**

We identified 16 and 125 patients with primary CMV and EBV infection, respectively (rates of 3.1 and 23.8 cases/year, respectively). Patients in the CMV group were older (median 34 vs. 22 years), had a longer illness duration before presentation (median 14 vs. 7 days) and more frequently systemic symptoms compared to patients in the EBV group. Increased lymphocyte count and presence of atypical lymphocytes were observed in both groups, yet less frequently and less pronounced in the CMV group. The overall number of performed tests (including laboratory and radiology tests) was significantly higher in the CMV group (median 11.5 vs. 3.0) before arriving at the final diagnosis. Antibiotic treatment was more frequently prescribed in patients with primary EBV infections (40 vs. 25%).

**Conclusions:**

Given its low incidence and non-specific symptoms, establishing a diagnosis of primary CMV infection can be challenging. Knowledge about clinical features of primary CMV infection in the immunocompetent host might help to adopt a stepwise approach to diagnosis avoiding over-testing.

## Introduction

Cytomegalovirus (CMV) is a member of the human Herpesviridae family, along with the Epstein-Barr virus (EBV), herpes simplex viruses 1 and 2, varicella-zoster virus und herpesviruses 6, 7, and 8 (1).

Global CMV seroprevalence is estimated to be 83% in the general population (2). CMV seroprevalence increases with age; it was found to be as low as 20.7% among 1–5 year-old children and reaches nearly 100% in the elderly populations of developing countries. Higher CMV prevalence rates are found in crowded and socially disadvantaged communities as well as in developing countries (3). The highest CMV seroprevalence was found in the World Health Organization (WHO) Eastern Mediterranean region (90%) and the lowest in the WHO European region (66%) (2). Infection is usually acquired early in life, during childhood or in young adulthood. Transmission occurs through exposure to saliva, urine, stool, breast milk, semen and other secretions from infected humans. Organ transplantation and blood transfusion are other potential transmission routes (3).

Infection induces the production of CMV-specific IgM and, later, IgG antibodies. IgG antibodies persist for life. Serological detection of IgM antibodies (+/- positive IgG antibodies) during an acute mononucleosis-like illness is usually sufficient for diagnosing primary CMV infection (1).

CMV causes a variety of clinical syndromes: Primary CMV infection in the fetus or the newborn and primary infection or reactivation in the immunocompromised host, may present as severe and potentially life-threatening disease (1, 3). In contrast, primary infection is often inapparent in the immunocompetent adult (1, 3). However, it may cause a non-specific, prolonged febrile illness, or a syndrome resembling infectious mononucleosis, called “mononucleosis-like syndrome” (MLI) by some authors (4). Fever, which may be prolonged up to 4 weeks, is described in nearly all symptomatic CMV patients. Other frequently observed symptoms are malaise, chills, headache, fatigue and a sore throat. Exudative pharyngitis, splenomegaly, cervical adenopathy, and a non-specific rash may also occur, yet are less frequently described compared to primary EBV infection [infectious mononucleosis (IM)]. Lymphocytosis [increase of 50 percent or more in the number of lymphocytes, with at least 10 percent atypical lymphocytes (4)] and increased liver function tests are frequently encountered laboratory changes. The presentation may resemble IM, making it difficult to distinguish the two viruses. Generally, the degree and frequency of these manifestations are milder in primary CMV compared to primary EBV infection (1, 4–7). Given the non-specific and non-focal symptoms of primary CMV establishing a diagnosis may be challenging (8), in particular in the setting of a tertiary care center where physicians are biased toward severely ill patients.

We performed a retrospective analysis of patients treated at our center over a 5-year period in whom a final diagnosis of primary CMV infection was established. We assessed their demographic, clinical, and laboratory characteristics and compared them to patients with primary EBV infection.

## Patients and Methods

This retrospective, monocentric study was conducted at the University Hospital Basel, Switzerland, a 700-bed academic tertiary care center with a catchment area of ~500,000 individuals.

We searched our hospital's laboratory database for patients with serology results compatible with either primary CMV infection or primary EBV infection who had been treated at our center between January 2016 and March 2021 (5.25 years). Eligible patients were defined as those aged ≥18 years and without an immunocompromising condition who were diagnosed with primary CMV or EBV infection. Exclusion criteria were: documented refusal of the general research consent for the use of routinely obtained personal and medical data, incomplete information about history and other important findings, other infections deemed to be responsible for the clinical picture, pregnancy, or any immunocompromising medical condition such as human immunodeficiency virus (HIV) infection, end stage renal failure, active hematologic or solid organ malignancy, hematopoietic stem cell transplantation (HSCT) or solid organ transplantation, or use of immunosuppressive therapy. The final diagnosis was ascertained and eligibility of patients determined by a board-certified internal medicine specialist and a dual-trained board-certified internal medicine/infectious diseases specialist taking into account the final diagnosis on the discharge summary, the history, clinical examination, laboratory and imaging results of the patients with a serology compatible with primary CMV or EBV infection.

We performed a detailed chart review to collect demographic, clinical and laboratory data. We further analyzed the number, nature, and consequences of the performed tests and treatments in these patients.

CMV- and EBV-specific antibody testing was performed using commercially available chemiluminescence immunoassay (CLIA) (CMV IgG and IgM: Immulite 2000, Siemens Healthineers, Germany; CMV IgG avidity, EBV VCA IgG and IgM, EBNA IgG: Liaison, DiaSorin, Italy).

The local ethics committee (Ethikkommission Nordwest- und Zentralschweiz, EKNZ) approved the study with a waiver of informed consent (EKNZ 2020-00611).

Continuous variables were reported as median [interquartile range (IQR)] and were compared using the Mann-Whitney U test, if not normally distributed or as mean +/- SD and were compared using the Student's *t*-test. Categorical variables were expressed as proportions and counts and compared using the Fisher's exact test. Tests were done at the 2-sided 5% significance level. All statistical analyses were performed with SPSS (Version 25, IBM, Chicago, Illinois, U.S.A.).

## Results

**Figure 1:**
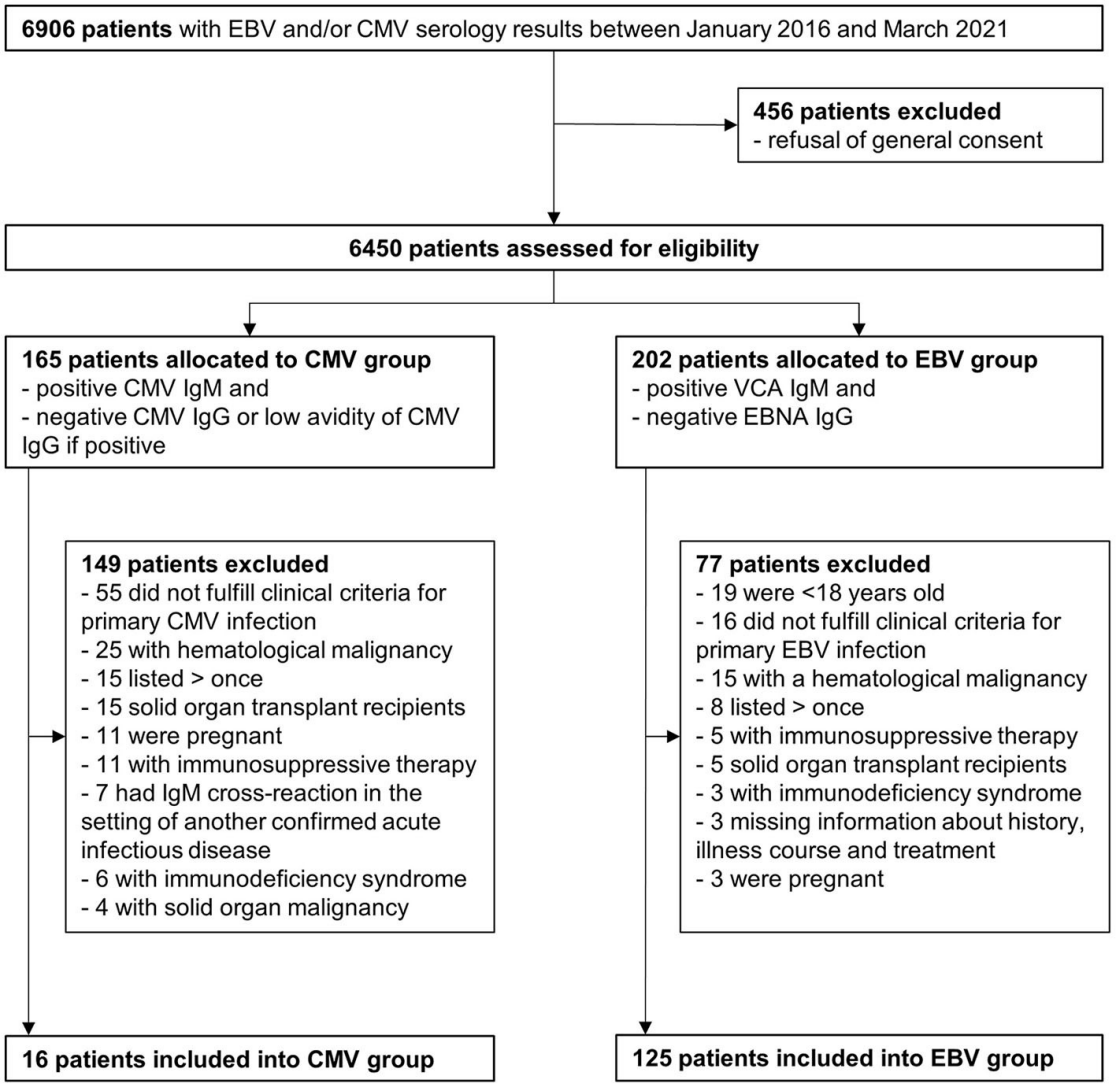
Flow diagram of the study.

We identified 6,906 patients with CMV and/or EBV serology results between January 2016 and March 2021. We excluded 456 patients with documented refusal regarding the use of their personal and clinical data for research purposes (Figure 1). Among the 6,450 patients assessed for eligibility, 165 satisfied our serology criteria for further assessment of primary CMV infection, and 202 patients had serology results consistent with primary EBV infection. In the CMV group, 149 patients were excluded because they met ≥1 exclusion criterion leaving 16 patients with primary CMV infection for the study (rate 3.1 cases/year). In the EBV group, 77 patients were excluded because they met ≥ 1 exclusion criterion, and hence 125 patients were included into the primary EBV infection group (23.8 cases/year).

### Primary CMV Infection

#### Patients

**Figure 2:**
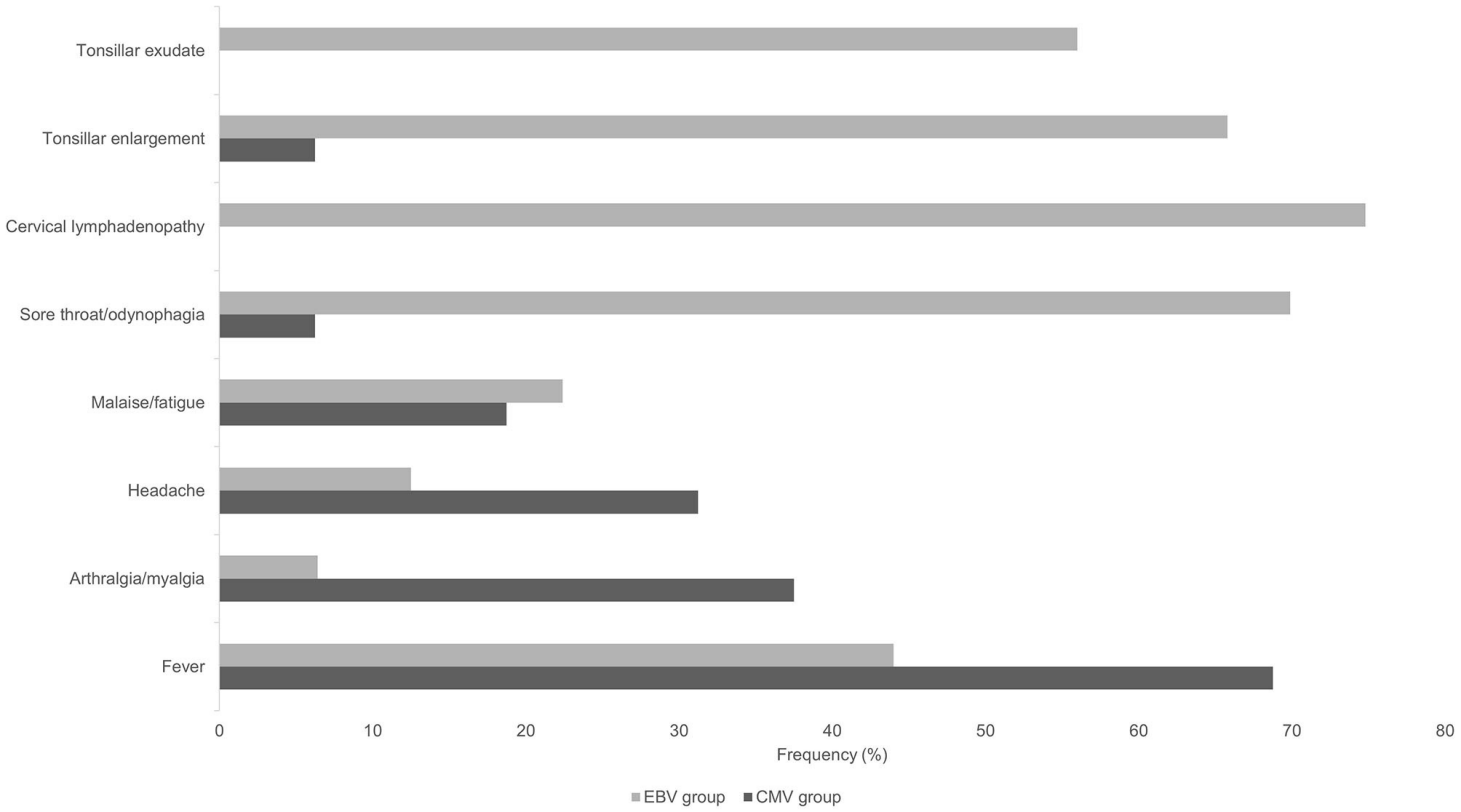
Frequency of symptoms and findings of clinical examination in patients with primary CMV and primary EBV infection.

Median age was 36.3 years (range 19–57 years) with an equal gender distribution. Median symptom duration before presentation was 14 days (range 5–42 days). The most frequently reported symptoms were fever (*n* = 11, 69%) and arthralgia (*n* = 6, 38%). Other symptoms including malaise, night sweats or diarrhea were less commonly reported. Clinical examination was unremarkable in the great majority of patients (*n* = 13, 81%), including a lack of lymphadenopathy. Typical IM symptoms and signs such as sore throat, tonsillar enlargement, tonsillar exudate and cervical adenopathy, were only rarely encountered in primary CMV infection (Figure 2). Laboratory analyses revealed an elevated absolute lymphocyte count and atypical lymphocytes in the majority of patients (50% and 88%, respectively). In addition, moderately elevated liver function tests were noted in all patients (Supplementary Table I), whereas C-reactive protein (CRP) concentrations were normal in 38% of the patients, and mildly elevated (10-60 mg/l) in 56%.

#### Management and Therapy

Fifteen patients (94%) had consulted at least one physician before presentation at our center including their general practitioner (GP, *n* = 13, 81%) and other specialists (neurologist, gastroenterologist etc.). Eight patients (50%) with primary CMV infection were admitted with a median length of stay of 2.5 days (range 1–16 days). For each patient, a median of 11.5 tests (range 2–25) in addition to hematology and biochemistry tests were performed.

Abdominal ultrasound was performed in 10/16 of patients (63%), showing splenomegaly in all cases. One additional patient had splenomegaly documented in an abdominal magnetic resonance imaging (MRI) performed before hospital referral. Chest X-rays were performed in 4/16 patients (25%), which were unremarkable except for one showing a potential consolidation. MRI and computed tomography (CT) scans were frequently utilized before admission (*n* = 7, 44%). All imaging studies yielded no significant findings.

Blood cultures (BC) were drawn in 7 patients (44%) yielding no growth. Additional serologies (median 5, range 0–10) were performed in nearly all patients (15/16 patients) including EBV (*n* = 15), human immunodeficiency virus (HIV) (*n* = 11), hepatitis B and C virus (HBV and HCV) (*n* = 10), hepatitis A virus (HAV) (*n* = 6), hepatitis E virus (HEV) (*n* = 4), parvovirus B19 (*n* = 4) and herpes simplex virus (HSV) (*n* = 3) serologies. Other frequently performed laboratory tests were: protein electrophoresis (*n* = 6), immunoglobulin levels (*n* = 5) and lymphocyte immunophenotyping (*n* = 3). All tests yielded no significant findings.

Four patients (25%) received empirical antibiotic therapy for suspected atypical pneumonia or streptococcal pharyngitis. Patient management details are listed in Supplementary Table II.

### Primary EBV Infection

#### Patients

Median age was 22.0 years (range 18–62 years) with an equal gender distribution. Median symptom duration before presentation was 7 days (range 1–42 days). The most frequently reported symptoms were sore throat (70%), fever (44%) and malaise/fatigue (22%). Cervical lymphadenopathy (74.8%), tonsillar enlargement (66%) and tonsillar exudate (56%) were notable examination findings. Laboratory analyses revealed an elevated absolute lymphocyte count and atypical lymphocytes in the majority of patients (85 and 100%, respectively). CRP was within the normal range or mildly elevated (10–60 mg/l) in 29 and 56% of the patients, respectively. Higher CRP values (>60 mg/l) were observed in 15% of the patients.

#### Management and Therapy

Seventy-one percent of the patients in the EBV group had consulted at least one physician before presentation at our university hospital, including their GP (58%), other emergency departments (15%) or specialists. Forty four percent of EBV patients were admitted with a median length of stay of 3.0 days (range 1–8 days). Fifty one percent were admitted to the general ward of the Internal Medicine Division, 45% to the otorhinolaryngology (ORL) Department. For each patient, a median of 3 tests (range 0–21) in addition to routine hematology and biochemistry laboratory were performed. Abdominal ultrasound was performed in 62%, showing splenomegaly in 73%. Chest X-rays and MRI/CT scans were performed in the minority of patients (<10%).

Blood cultures were drawn in one third of the patients yielding no growth. Additional serologies were performed in 58% of the patients (median 1, range 0–11), including HIV (78%), CMV (69%), HBV (60%) and HCV (56%). All tests yielded no significant findings.

Empirical antibiotic therapy was prescribed in 39.8% of the EBV patients. Among patients cared for by the ORL Department, 93% received antibiotic therapy compared to 10% in patients treated by other departments. Other treatments included needle aspiration (*n* = 3), abscess drainage or tonsillectomy (*n* = 3), high dose corticosteroids because of respiratory compromise due to severely enlarged tonsils (*n* = 2), and embolization of splenic vessels due to spontaneous spleen rupture (*n* = 1). Management and complications in the EBV group are listed in Supplementary Table III.

### Differences in Primary CMV vs. Primary EBV Infection

Patients in the primary CMV group were older (median 34 vs. 22 years in EBV patients), had a longer symptom duration (median 14 vs. 7 days), and symptoms most frequently reported were systemic (fever, arthralgia, headache and malaise/fatigue), whereas the most frequent complaint in the EBV group was focal (sore throat in 70%). Clinical examination was unremarkable or non-specific in all CMV patients, whereas the typical presentation of EBV-IM (cervical lymphadenopathy, enlarged tonsils and tonsillar exudate) was noted in the majority of EBV patients (Figure 2).

Regarding laboratory work-up, the following significant differences were noted: patients in the CMV group showed lower absolute lymphocyte and large unstained cell (LUC) counts, lower lymphocyte/WBC and LUC/WBC ratios, and a lower percentage of atypical lymphocytes (Table 1). Of note, only 50% of the patients in the CMV group had an elevated lymphocyte count, whereas this proportion was 85% in the EBV group. While liver function tests were elevated at a similar degree, there was a trend toward higher CRP values in EBV patients (median 13.7 vs. 19.3 mg/l, *p* = 0.08). The overall number of performed tests was significantly higher in the CMV group (median 11.5 vs. 3.0).

**Table 1:** Comparison of patients' characteristics, history, laboratory results and management of primary CMV vs. primary EBV infections. The data are reported as median (range) or n (%).

	**CMV group (*n* = 16)**	**EBV group (*n* = 125)**	***P*-value**
Age, years	34.0 (19–57)	22.0 (18–62)	**<0.001**
Female patients	8 (50)	60 (48)	1.000
Symptoms and signs	Fever: 11 (68.75) Arthralgia/myalgia: 6 (37.5) Headache: 5 (31.25) Malaise/fatigue: 3 (18.75) Sore throat/odynophagia: 1 (6.25) Cervical lymphadenopathy: 0 (0)	Cervical lymphadenopathy: 92/123 (74.8) Sore throat/odynophagia: 87/125 (69.90) Tonsiliits: 81/123 (65.8) Fever: 55/125 (44) Malaise/fatigue: 28/125 (22.4) Nausea: 17/125 (13.6) Headache: 16/125 (12.5) Abdominal discomfort: 12/125 (9.6) Rash: 11/125 (8.8) Arthralgia/myalgia 8/125 (6.4)	-
Symptom duration before presentation, days	14 (5–42)	7 (1–42)	**<0.001**
**Laboratory data**			
Lymphocyte count (0.9–3.0 G/l)	2.93 (0.26–7.9)	5.3 (0.71–18.4)	**0.002**
Lymphocytes/WBC (<48%)	46.35 (4.2–65)	51.9 (16–69.9)	**0.047**
LUC count (0–0.31 G/l)	0.60 (0.08 –.71)	1.0 (0.1–12.4)	**0.020**
LUC/WBC (0–4.0%)	5.75 (1.2–28)	8.8 (0.85–31)	**0.049**
AL/WBC (%)	7.0 (0–15)	12.0 (1.5–36.5)	**0.003**
ASAT (<34 IU/l)	103 (22–184)	103 (22–724)	0.344
ALAT (<59 IU/l)	155 (23–374)	165 (16–1663)	0.526
CRP (<10 mg/l)	13.7 (1–91)	19.3 (0.5–221)	0.081
Patients hospitalized	8 (50)	55 (44)	0.791
*Internal Medicine*	6/8 (75)	28/55 (50.9)	
*ORL*	0	25/55 (45.45)	
*Other*	2/8 (25)	2/55 (3.63)	
Inpatient length of stay, days	2.5 (1–16)	3.0 (1–8)	0.515
Number of additional tests	11.5 (2–25)	3.0 (0–21)	**<0.001**
Number of additional serologies	5 (0–10)	1 (0–11)	**0.005**
Abdominal ultrasound	10 (62.5)	78 (62.4)	0.611
Splenomegaly	11/11* (100)	57/78 (73)	
Antibiotic treatment	4/16 (25)	49/123 (39.8)	0.054

WBC, white blood cells; LUC, large unstained cells; AL, atypical lymphocytes; ASAT, aspartate aminotransferase; ALAT, alanin aminotransferase; CRP, C-reactive protein; ORL, otorhinolaryngology. ^*^One patient had abdominal magnetic resonance imaging (MRI) with documentation of splenomegaly before hospital presentation. The bold values indicates the values of p < 0.05 which are statistically significant.

All but one primary CMV patient (94%) had consulted another physician before presenting at our center; this proportion was lower in the EBV group (71%). However, in the CMV group, the proportion of patients who were prescribed empirical antibiotic therapy was lower (25 vs. 40%).

## Discussion

We retrospectively investigated demographic and clinical characteristics and the management of patients who were diagnosed with primary CMV infection compared to primary EBV infection in a tertiary care center in Switzerland over a 5-year period.

Primary CMV infection was infrequently diagnosed at our tertiary care center in contrast to primary EBV infection (3.05 cases/year, vs. 23.81 cases/year). This finding is in line with previous studies. The annual rate of primary CMV infections in retrospective studies conducted at tertiary care centers ranged from 2–4 cases/year (5, 6, 8, 9). A higher rate of primary CMV infections among immunocompetent adults was only diagnosed in the UK when performing CMV serologies routinely in all patients presenting to their GP or two hospitals with compatible symptoms: 126 patients over a 2.5-years period (7.2 cases/year). However, 85% of the patients in this case series presented to their GP (7). This higher number of identified CMV cases in a population where all symptomatic patients were systematically tested for CMV underscores the fact that primary CMV infection is underdiagnosed if not routinely screened for by GPs.

A large analysis conducted between 2015 and 2019 showed overall CMV seroprevalences among blood cell donors of 30% in Germany and 32% in the United Kingdom, whereas seroprevalences were above 60% in Poland and Chile (10). These results imply that CMV seroprevalence has declined or has been overestimated in resource-rich countries in previous studies. The changing epidemiology of CMV delays the age of acquisition, which may increase the likelihood of a symptomatic infection when acquired at a higher age (11, 12). In fact, 25% of patients diagnosed with primary CMV infection in the present study were older than 50 years. Hence, primary CMV infection should be considered in the differential diagnosis even in older patients.

The present cohort's prototypic patient with primary CMV infection may be characterized as approximately 35–40 years old, referred by his GP because of fever of unknown origini (FUO) for 2 weeks with no local signs on clinical examination, a mild lymphocytosis with presence of atypical lymphocytes, and moderately elevated liver function tests whereas inflammation is only mildly present. This is exemplified by a patient that presented to our hospital shortly after termination of the inclusion period for this study (further details of this patient are found in Supplementary Table IV). The non-specific, non-focal symptoms observed in primary CMV infection may complicate or at least hinder prompt recognition and diagnosis, as observed in our patients. A majority of primary CMV patients in the present study consulted multiple physicians and underwent extensive testing before the final diagnosis was established in contrast to patients with primary EBV infections (median number of tests per patient 11.5 vs. 3.0). The high proportion of patients referred to our center by their GP illustrates that identifying this disease is challenging in a primary care setting, even though primary care physicians might be more familiar with the disease because they are more frequently confronted with it. In tertiary care hospitals physicians are biased toward complex patients and severe diseases. Crowded emergency departments, expectations of referring physicians or patients, liability issues and the lack of knowledge about the non-specific clinical picture of primary CMV infection may contribute to extensive and immediate testing and even therapy for a “simple” viral infection early upon presentation in a tertiary care setting, as our case series exemplifies. Knowledge about its presentation characteristics (prolonged fever, moderate elevation of lymphocytes, presence of atypical lymphocyte and moderate elevation of liver function tests) might help to adopt a stepwise approach to diagnosis. In our opinion, testing for CMV, EBV (because of its similar clinical and laboratory presentation) and HIV (because of its similar presentation and enormous socioeconomic burden) is a first step, and, only if these initial serologies are negative, additional investigations should be pursued. In the age of “smarter medicine” and given the imperative to curb health care expenditures, this stepwise approach seems to constitute a reasonable path to follow when approaching these patients.

Our findings regarding the characteristics of primary CMV infection mirror results from previous studies. For example, primary CMV infection patients' age ranged from 32.5 to 38.6 years in three studies (5, 6, 9) compared to 34 years in our analysis. The most frequently reported symptoms were non-specific and similar to our findings: fever, headache, fatigue, malaise and sweats (5–9). Symptom duration before presentation was identical to our findings (14 days) in a Japanese study that included inpatients and outpatients with IM symptoms (5), while Nolan et al. found a longer symptom duration of 28 days before presentation among FUO patients who were admitted to a tertiary care center in the US (8). Compared to primary EBV infection, patients with primary CMV infection were older and reported non-specific symptoms of a longer duration compared to the local and classical symptoms of primary EBV patients. However, primary EBV infection in older adults was also reported to be associated with prolonged fever and hepatitis rather than the typical triad of lymphadenopathy, pharyngitis and fever (13). In addition, atypical lymphocytosis or elevation of lymphocyte/WBC ratio was less pronounced in primary CMV infection, which is in agreement with two previous studies (5, 9).

While patients in the EBV group underwent less testing before being correctly diagnosed, they were prescribed empirical antibiotic therapy more frequently. Antibiotic prescription strategies varied among the treating clinics, with higher prescription rate among patients treated by the ORL Department. All patients hospitalized in the ORL Department had a documented history of sore throat and/or odynophagia, but only few patients required surgical treatment or corticosteroids because of upper airway compromise in contrast to patients hospitalized in the other departments, where no complications were reported. These observations suggest that EBV patients treated by the ORL Department had more complicated disease courses. In the literature, 1.5–6% of patients with peritonsillar abscess (PTA) are described to have primary EBV infection (14–18), and one percent of patients with primary EBV infection develop PTA (19, 20). The role of antibiotic therapy to prevent complications, especially PTA, in primary EBV infection patients is unclear (21, 22), and to our knowledge, there is no evidence that antibiotics may prevent complications in this setting.

### Strengths and Limitations

By using broad serological inclusion criteria, the present study gives a good overview of the number of patients with primary CMV or EBV infection leading to consultation or admission in a tertiary care hospital. However, our study has a number of limitations. First, the number of primary CMV infection patients assessed was small. Although our results are similar to those of other studies, caution is necessary in the generalization of CMV patients' characteristics. Some of the differences observed between the two groups might be explained solely by the small numbers. Second, a systematic testing of all patients for primary CMV disease with a compatible clinical picture is not done in our hospital, and hence we may have underestimated the number of primary CMV cases at our hospital. However, a policy of generously ordering serologies (including CMV serology) in case of FUO or patients with non-focal symptoms as observed in primary CMV patients, probably means that not many cases will have been missed in our hospital. Third, our data was extracted from notes of other providers, and hence assessment of performed tests and treatments before presentation at our hospital was only partially possible because of a lack of documentation in the patients' charts. This also includes the duration and nature of symptoms and information about the possible way of transmission.

## Conclusion

Primary CMV infections are rarely diagnosed in tertiary care hospitals. Because of its relatively low frequency and unspecific clinical presentation, primary CMV infection in the immunocompetent adult host can be a challenging diagnosis. Knowledge about its presentation characteristics (prolonged non-specific symptoms such as fever, moderate elevation of lymphocytes, presence of atypical lymphocyte and moderate elevation of liver function tests) might help to adopt a stepwise approach to diagnosis and to avoid unnecessary diagnostic testing and treatment resulting in over-testing and increased health care expenditures.

## Data Availability Statement

The raw data supporting the conclusions of this article will be made available by the authors, without undue reservation.

## Author Contributions

SE and MO had full access to all of the data in the study and take responsibility for the integrity of the data and the accuracy of the data analysis. SE collected the data and prepared a first manuscript draft. All authors contributed substantially to the study design and interpretation, analyzed the data, contributed substantially to the writing of the manuscript, critically revised the manuscript for important intellectual content, and approved the final version.

## Conflict of Interest

The authors declare that the research was conducted in the absence of any commercial or financial relationships that could be construed as a potential conflict of interest.

## Publisher's Note

All claims expressed in this article are solely those of the authors and do not necessarily represent those of their affiliated organizations, or those of the publisher, the editors and the reviewers. Any product that may be evaluated in this article, or claim that may be made by its manufacturer, is not guaranteed or endorsed by the publisher.

## Abbreviations

CMV: Cytomegalovirus
EBV: Epstein-Barr Virus
IM: infectious mononucleosis
HIV: human immunodeficiency virus
HAV: hepatitis A virus
HBV: hepatitis B virus
HCV: hepatitis C virus
WBC: white blood cells
LUC: large unstained cells
ASAT: aspartate aminotransferase
ALAT: alanine aminotransferase
CRP: C-reactive protein
GP: general practitioner
ORL: otorhinolaryngology
FUO: fever of unknown origin
PTA: peritonsillar abscess.

## References

[1] Taylor GH. Cytomegalovirus. Am Fam Physician. (2003) 67:519–24+26. 12588074

[2] Zuhair M Smit GSA Wallis G Jabbar F Smith C Devleesschauwer B. Estimation of the worldwide seroprevalence of cytomegalovirus: a systematic review and meta-analysis. Rev Med Virol. (2019) 29:e2034. 10.1002/rmv.2034 30706584

[3] Dioverti MV Razonable RR Cytomegalovirus. Microbiol Spectr. (2016) 4. 10.1128/microbiolspec.DMIH2-0022-2015 27726793

[4] Hurt C Tammaro D. Diagnostic evaluation of mononucleosis-like illnesses. Am J Med. (2007) 120:911.e1–8. 10.1016/j.amjmed.2006.12.011 17904463

[5] Ishii T Sasaki Y Maeda T Komatsu F Suzuki T Urita Y. Clinical differentiation of infectious mononucleosis that is caused by Epstein-Barr virus or cytomegalovirus: a single-center case-control study in Japan. J Infect Chemother. (2019) 25:431–6. 10.1016/j.jiac.2019.01.012 30773381 PMC7128249

[6] Just-Nübling G Korn S Ludwig B Stephan C Doerr HW Preiser W. Primary cytomegalovirus infection in an outpatient setting–laboratory markers and clinical aspects. Infection. (2003) 31:318–23. 10.1007/s15010-003-3129-y 14556056

[7] Wreghitt TG Teare EL Sule O Devi R Rice P. Cytomegalovirus infection in immunocompetent patients. Clin Infect Dis. (2003) 37:1603–6. 10.1086/379711 14689339

[8] Nolan N Halai UA Regunath H Smith L Rojas-Moreno C Salzer W. Primary cytomegalovirus infection in immunocompetent adults in the United States - A case series. IDCases. (2017) 10:123–6. 10.1016/j.idcr.2017.10.008 29159070 PMC5684088

[9] Batalla AS Benito D Baumard S Brodard V Servettaz A Jaussaud R. et al. [Epstein-Barr virus and cytomegalovirus primary infections: a comparative study in 52 immunocompetent adults]. Med Mal Infect. (2011) 41:14–9. 10.1016/j.medmal.2010.07.012 20832213

[10] Behrens GA Brehm M Groß R Heider J Sauter J Baier DM. Noninvasive determination of CMV serostatus from dried buccal swab samples: assay development, validation, and application to 1.2 million samples. J Infect Dis. (2021) 224:1152–9. 10.1093/infdis/jiaa067 32052845 PMC8514182

[11] Wreghitt T Behr S Hodson J Irwin D. Feverish granny syndrome. Lancet. (1995) 346:1716. 10.1016/S0140-6736(95)92889-8 8551869

[12] Lancini D Faddy HM Flower R Hogan C. Cytomegalovirus disease in immunocompetent adults. Med J Aust. (2014) 201:578–80. 10.5694/mja14.00183 25390262

[13] Auwaerter PG. Infectious mononucleosis in middle age. JAMA. (1999) 281:454–9. 10.1001/jama.281.5.454 9952206

[14] Arkkila E Sipilä J Laurikainen E Suonpää J. Peritonsillar abscess associated with infectious mononucleosis. ORL J Otorhinolaryngol Relat Spec. (1998) 60:159–63. 10.1159/000027586 9579361

[15] Ahmad UA Anari S. Infectious mononucleosis screening in quinsy patients. Eur Arch Otorhinolaryngol. (2010) 267:113–5. 10.1007/s00405-009-0969-9 19340442

[16] Hanna BC McMullan R Gallagher G Hedderwick S. The epidemiology of peritonsillar abscess disease in Northern Ireland. J Infect. (2006) 52:247–53. 10.1016/j.jinf.2005.07.002 16125782

[17] Ryan C Dutta C Simo R. Role of screening for infectious mononucleosis in patients admitted with isolated, unilateral peritonsillar abscess. J Laryngol Otol. (2004) 118:362–5. 10.1258/002221504323086552 15165311

[18] Shareef MM Balaji N Adi-Romero P. Screening for glandular fever in patients with Quinsy: is it necessary? Eur Arch Otorhinolaryngol. (2007) 264:1329–31. 10.1007/s00405-007-0355-4 17569070

[19] Johnsen T. Infectious mononucleosis and peritonsillar abscess. J Laryngol Otol. (1981) 95:873–6. 10.1017/S0022215100091544 6943240

[20] Johnsen T Katholm M Stangerup SE. Otolaryngological complications in infectious mononucleosis. J Laryngol Otol. (1984) 98:999–1001. 10.1017/S0022215100147875 6593388

[21] Klug TE. Peritonsillar abscess: clinical aspects of microbiology, risk factors, and the association with parapharyngeal abscess. Dan Med J. (2017) 64:B5333. 28260599

[22] Danstrup CS Klug TE. Low rate of co-infection in complicated infectious mononucleosis. Dan Med J. (2019) 66:A5564. 31495372

