# Supplementary material for: Challenges of Primary Care Medicine in a Tertiary Care Setting—The Case of Primary CMV Infection Compared to Primary EBV Infection: A Retrospective Cohort Study
Source: Front Med (Lausanne). 2022 Jun 14;9:880610. doi: 10.3389/fmed.2022.880610 (PMC9239529; doi:10.3389/fmed.2022.880610)
Supplement: Supplementary file 1 [file Data_Sheet_1.docx]

**Supplemental material**

**Supplement table I**: Characteristics of patients with primary CMV infection including symptoms, clinical examination findings and laboratory findings upon presentation. Patients A-H were inpatients, patients G-P were outpatients.

| Patient | Sex, age | History | Clincial examination findings | Abdominal sonography performed^§^ | Lymphocytes (G/l)  [0.9-3.0] | Ly/WBC  (%)  [19-48] | LUC (G/l)  [0-0.31] | LUC/WBC (%)  [0 - 4.0] | AL/WBC (%) | ASAT (IU/l)  [11 - 34] | ALAT (IU/l)  [9 - 59] | CRP (mg/l)  [<10] |
| --- | --- | --- | --- | --- | --- | --- | --- | --- | --- | --- | --- | --- |
| A | f, 50 | Fever, anorexia, cephalgia,arthralgia, malaise for 3 weeks. | Unremarkable. | Yes | 2.9 | 45 | **0.64** | **10** | **12** | **136** | **162** | **23** |
| B | f, 57 | Fever and cephalgia for 1 week, cough. | Unremarkable. | No | 1.5 | 23 | 0.21 | **4** | - | **37** | **48** | **29** |
| C | m, 33 | Fever for 3 weeks, night sweats, 3 kg weight loss, abdominal cramps. | Inguinal lymphadenopathy | Yes | **7.9** | 52 | **1.02** | **7** | **4** | **112** | **165** | **17** |
| D | f, 20 | Fever, palpitations and dyspnea for 5 days. | Unremarkable. | No | 2.3 | 39 | **0.85** | **14** | **8** | **79** | **138** | 6 |
| E | m, 31 | Fever for 2 weeks. | Unremarkable. | Yes | **6.6** | **65** | **1.2** | **12** | **4** | **136** | **199** | **13** |
| F | m, 32 | Fever, lower back pain for 1 week. | Unremarkable. | No | **5.7** | **48** | **0.67** | **5** | **6** | **141** | 1**80** | **28** |
| G | f, 37 | Gingivial bleeding, onset 1 week after symptoms of a common cold. | Petechia and hematomas | Yes | 2.5 | **49** | 0.23 | **5** | **7** | **64** | **78** | 4 |
| H | f, 21 | Cephalgia, burning skin sensation, sweating and diffuse abdominal pain for 2 weeks. | Unremarkable. | Yes | **7.4** | **58** | **1.1** | **9** | **10** | **100** | **137** | **91** |
| I | m, 28 | Fever, night sweats, arthralgia for 2 weeks, cough for 2 days. | Unremarkable. | Yes | **3.7** | **51** | **0.4** | **6** | - | **161** | **397** | **14** |
| J | f, 43 | Fatigue for several days. | Unremarkable. | No | 2.1 | 47 | 0.19 | **4** | **15** | **67** | **82** | 7 |
| K | m, 44 | Fever and arthralgia for 2 weeks, transient diarrhea. | Unremarkable. | Yes | **4.0** | 44 | **0.81** | **9** | **10.5** | - | **154** | **27** |
| L | f, 19 | Fatigue, malaise, anorexia and cervical lymphadenopathy | Unremarkable. | No^§§^ | 1.8  - | 26  - | 0.08  - | 1  - | 0  - | 22  **46*** | 23  **53*** | 1  - |
| M | f, 51 | Fever, malaise and transient diarrhea.for 3 weeks. | Unremarkable. | No | 2.5 | 45 | **0.33** | **6** | 0 | **55** | **87** | 2 |
| N | m, 52 | Fever, malaise, sore throat, arthralgia and cephalgia for 4-6 weeks. | Tonsilar enlargement | Yes | **3.0** | 39 | 0.21 | 3 | - | **184** | **313** | **11** |
| O | m, 28 | Cephalgia and dizziness for 2 weeks. | Unremarkable. | Yes | 0.3 | 4 | **1.71** | **28** | - | - | **293** | 5 |
| P | m, 35 | Fever for two weeks, dark urine for 1 week. | Unremarkable. | Yes | **5.2** | **51** | **0.57** | **6** | **7** | **106** | **156** | **19** |

**Abbreviations**: laboratory findings listed as follows: (analysis, (units), [normal range]), WBC: white blood cells, LUC: large unstained cells, AL: atypical lymphocytes. ASAT: aspartate aminotransferase, ALAT: alanin aminotransferase, CRP: C-reactive protein. Results out of the normal range are highlighted in bold.

*performed at symptom onset at primary practitioner

^§^splenomegaly was documented in all patients who had abdominal sonography; ^§§^splenomegaly documented in abdominal magnetic resonance imaging before hospital referral.

|  | Consultation before presentation at our center | Tests and treatments performed before presentation at our center | Length of stay (days) | Serologies | Other laboratory tests | Timing of CMV serology | Imaging | Treatment |
| --- | --- | --- | --- | --- | --- | --- | --- | --- |
| A | yes: GP | - | 5 | EBV, HIV, HAV, HBV, HCV, HEV | BC, EBV PCR, CMV PCR, M protein, free light chains, stool cultures, Malaria (blood smear light microscopy analysis repeated 3 times) | Day 3 | Abdominal US  Chest radiography | Ceftriaxone 2 days on admission day and hospitalization day 1 (FUO) |
| B | yes: GP | - | 16 | EBV, Mycoplasma pneumoniae | BC, respiratory pathogens panel PCR (nasopharyngeal swab), urine legionella and pneumococcal antigen test, sputum cultures | Day 9 | Chest radiography  Head MRI | Amoxicillin/clavulanic acid 2 days, levofloxacin 13 days (suspected atypical pneumonia) |
| C | yes: GP | - | 3 | EBV, HIV | BC, IgA, IgG, IgM, FACS (lymphocyte immunophenotyping) | Day 0 | Chest and abdomen CT-scan | - |
| D | yes: ED of other hospital, neurologist | - | 1 | EBV, HIV, HBV, HCV, Toxoplasma gondii | BC, syphilis screening, Troponin, D-dimers | Day 0 | CTPA  transthoracic echocardiography  stress echocardiography | - |
| E | yes: GP | - | 2 | EBV, HIV, HAV, HBV, HCV, Chlamydia trachomatis | BC, syphilis screening, Troponin | Day 1 | Abdominal US | - |
| F | yes: GP | Spine MRI, amoxicillin-clavulanic acid 5days | 5 | EBV, HIV, HAV, HBV, HCV, Parvovirus B19 | BC, Chlamydia trachomatis PCR, Neisseria gonorrhea PCR, T-SPOT.TB, rheumatoid factor, anti-CCP-antibodies, M protein | Day 0 | Chest radiography | - |
| G | yes: GP | - | 2 | EBV, HIV, HBV, HCV, Parvovirus B19 | M protein | Day 0 | Abdominal US | Dexamethasone, IVIG (ITP secondary to CMV infection) |
| H | yes: GP | HIV, HBV, HCV, Syphilis screening | 1 | EBV, HSV I+II | M protein, IgA, IgG, IgM | Day 1 | Abdominal US  Head MRI | - |
| I | yes: GP | - | outpatient | EBV | - | First visit | Abdominal US  CTPA | - |
| J | yes: gynecologist | - | outpatient | EBV, Mycoplasma pneumoniae, Parvovirus B19 | Coombs test, FACS (lymphocyte immunophenotyping), IgA, IgG, IgM | Second visit | - | RBC transfusion (4 units) (hemaloytic anemia secondary to CMV infection) |
| K | no | - | outpatient | EBV, HIV, HBV, HCV, Syphilis, Toxoplasma gondii | - | First visit | Abdominal US | - |
| L | yes: GP, gastroenterologist | Abdominal MRI, M protein, free light chains | outpatient | EBV | IgA, IgG, IgM, free light chains, M protein, ANA | First visit | - | - |
| M | yes: GP | - | outpatient | - | CMV PCR, FACS (lymphocyte subpopulations) | First visit | - | - |
| N | yes: GP | Penicillin 10 days | outpatient | EBV, HIV, HAV, HBV, HCV, HEV | Malaria (blood smear light microscopy analysis repeated 3 times) | Second visit | Abdominal US  Chest radiography | - |
| O | yes: GP | HAV, HBV, HCV | outpatient | EBV, HIV, HEV, HSV I + II, HHV-6, HHV-7, Parvovirus B19 | Alpha-1 antitrypsin, ceruloplasmin, IgA, IgG, IgM, IgG subclasses, M protein, ANA, ANCA, anti-MPO, anti-PR3, anti-LKM-1 IgG, anti-parietal celle IgG | Second visit | Abdominal US | - |
| P | yes: urologist, GP | - | outpatient | EBV, HIV, HAV, HBV, HCV, HDV, HEV, HSV I+II , Tick-borne encephalitis, Mumps | BC, T-SPOT.TB | First visit | Abdominal US | - |

**Supplement table II**: Physician visits, diagnostic procedures before presentation at our tertiary care center, treating clinic, length of stay and performed diagnostic testing in CMV patients. All test results were normal.

**Abbreviations:** GP: general practitioner, ED: Emergency Department, MRI: magnetic resonance imaging, CT: computed tomography, CTPA: computed tomography pulmonary angiography, BC: blood cultures, HAV: hepatitis A virus, HBV: hepatitis B virus, HCV: hepatitis C virus, HEV: hepatitis E virus, EBV: Epstein-Barr virus, HSV: herpes simplex virus, HIV: human immunodeficiency virus, HHV: Human herpes virus, T-SPOT.TB: enzyme-linked immune absorbent spot (ELIspot) for tuberculosis diagnosis (Oxford Immunotec), FACS: fluorescence activated cell sorting, M protein: serum protein immunofixation electrophoresis, anti-CCP-antibodies: anti-cyclic citrullinated peptide antibodies, ANA: anti-nuclear antibodies, ANCA: anti-neutrophil cytoplasmic antibodies, anti-MPO: anti-myeloperoxidase antibodies, anti-PR3: anti-proteinase 3 antibodies, anti-LKM-1: anti-liver-kidney microsomal 1 antibodies, US: ultrasonography, FUO: fever of unknown origin, IVIG: intravenous immune globulin, ITP: immune thrombocytopenia

**Supplement table III:** Management characteristics of EBV patients.

|  | Inpatients (44%, n=55) | | | Outpatients (56%, n=70) |
| --- | --- | --- | --- | --- |
| Treating clinic | ORL 45.45% (n=25)  IM 50.9% (n=28)  General surgery: (n=1)  Dermatology (n=1) | | | ED 42.85% (n=30)  ORL 28.75% (n=20)  IM 28.75% (n=20) |
| Length of stay, days | Mean  3.38  ORL 3.3636  Other 3.77 | Median  3.0  3.0  4.0 | Range  1 – 8  1 – 8  1 - 8 |  |
| Abdominal sonography, % (n) | Not performed: 25.45 (14)  Performed, unremarkable: 23.63 (13)  Performed, showed splenomegaly: 49.09 (27)  Performed, showed signs of cholecystitis: 1.81 (1) | | | Not performed: 47.14 (33)  Performed, unremarkable: 10 (7)  Performed, showed splenomegaly: 42.85 (30)  Performed, showed signs of cholecystitis: 0 |
| Blood cultures | 49.09 (27) |  |  | 20.0 (14) |
| Patients with antibiotic prescription, % (n) | 50.9 (28) |  |  | 27.9 (19/68) |
| Operations | 3 Needle aspiration (no pus)  2 tonsillectomies  1 peritonsillar abscess drainage | | |  |
| Interventions | 1 gastroscopy and colonoscopy (suspicion of IBD)  1 embolization of splenic vessels because of spontaneous spleen rupture  1 lumbar puncture because of suspected GBS  1 skin biopsy (skin rash) | | |  |
| Other therapy | 2 iv methylprednisolone therapies (respiratory compromise)  2 patients treated with steroids and clemastine because of skin rash (not identified as of viral origin)  1 anticoagulation because of DVT | | |  |
| Adverse events | 1 allergic reaction to antibiotics | | |  |

**Abbreviations:** IM: Internal Medicine Department, ED: Emergency Department, ORL: Othorhinolaryngology Department, IBD: inflammatory bowel disease, GBS: Guillain-Barré syndrome, iv: intravenous, DVT: deep vein thrombosis.

**Supplement table IV**: Illness course of a 27-year-old male patient with primary CMV infection.

|  | Day -1  IM outpatient clinic presentation | Day 1  ED presentation and hospitalization | Day 3  Hospital discharge | Day 6  Planed followup visit | Day 10  ED visit | Day 17  Planned follow up visit |
| --- | --- | --- | --- | --- | --- | --- |
| Symptoms | Headache, recurrent fever night sweats for 5 days | Persistence of symptoms, arthralgia and lower back pain |  | Persistence of headache. Resolution of fever.  Fatigue. | New episode of fever, persistence of headache and arthralgia | Persistence of fever, night sweats, headache |
| Laboratory |  |  |  |  |  |  |
| Ly (G/l, 0.9 – 3.3) | 2.08 | 2.44 | 2.76 | **5.46** | **4.97** | **5.43** |
| Ly/WBC (%, 19- 48) | 40.3 | 42.2 | **48.6** | **58.5** | **55.2** | **60.9** |
| LUC (G/l, 0 – 0.310) | 0.25 | **0.31** | **0.33** | **0.44** | **0.50** | 0.3 |
| LUC/WBC (%, 0 – 4) | **4.8** | **5.3** | **5.8** | **4.7** | **4.6** | 3.4 |
| AL/WBC (%) | - | - | **3.0** | **10** | **8** | **2.5** |
| ASAT (U/l, 11 – 34) | - | **116** | **130** | **178** | **95** | **54** |
| ALAT (U/l, 9-59) | **61** | **120** | **178** | **228** | **180** | **95** |
| CRP (mg/l, <10) | 3.9 | 4.5 | 4.5 | 3.9 | 9.9 | 1.5 |
| Working hypothesis  Diagnosis | Tension headache  FUO | FUO and elevated transaminase of unclear origin  Meningitis | **Primary CMV infection** |  |  |  |
| Tests |  | - CT head  - Lumbar puncture, PCR panel, culture  - Blood cultures: 5 pairs  - SARS-CoV2 PCR (nasopharyngeal swab) | - US abdomen: splenomegaly  - **CMV serologies:**  **IgM positive**  **IgG positive, avidity <.15 (range: <.25)**  - EBV  - HAV, HBV, HCV, HEV  - Syphilis  - Free light chains, M protein  - Complement C3, C4  - RF, ANA, anti-mitochondrial IgG, anti- smooth muscle IgG, Anti-LKM-1 IgG, Anti-soluble liver antigen/liver pancreas IgG |  | SARS-CoV2 PCR (nasopharyngeal swab)  Urinalysis  Urine culture |  |

**Abbreviations**: IM: Internal Medicine Department, ED: Emergency Department. FUO: fever of unknown origin, US: ultrasonography, RF: rheumatoid factor, ANA: anti-nuclear antibodies, anti-LKM-1 IgG: anti-liver-kidney microsome type 1 IgG.
